# Effectiveness and Safety of a Clonidine Adhesive Patch for Children With Tic Disorders: Study in a Real-World Practice

**DOI:** 10.3389/fneur.2020.00361

**Published:** 2020-05-08

**Authors:** Chunsong Yang, BingYao Kang, Dan Yu, Li Zhao, Lingli Zhang

**Affiliations:** ^1^Department of Pharmacy, Evidence-Based Pharmacy Center, West China Second Hospital, Sichuan University, Key Laboratory of Birth Defects and Related Diseases of Women and Children (Sichuan University), Ministry of Education, Chengdu, China; ^2^Department of Health Policy and Management, West China School of Public Health, and West China Fourth Hospital, Sichuan University, Chengdu, China; ^3^Department of Pediatric Clinic, West China Second Hospital, Sichuan University, Chengdu, China; ^4^Department of Children's Genetic Endocrinology and Metabolism, West China Second Hospital, Sichuan University, Key Laboratory of Birth Defects and Related Diseases of Women and Children (Sichuan University), Ministry of Education, Chengdu, China

**Keywords:** effectiveness, safety, clonidine adhesive patch, tic disorders, real-world study

## Abstract

**Background:** Real-world evidence includes data from retrospective/prospective observational studies and observational registries, and provides insights beyond those addressed by randomized controlled trials. This study aimed to evaluate the effectiveness and safety of a clonidine adhesive patch (CAP) for children with tic disorder (TD) in a real-world setting (RWS).

**Methods:** This was an open-label, non-interventional, post-marketing, observational study in a RWS. Children diagnosed with TDs were enrolled from a pediatric neurology clinic in China, and the change in tic symptom severity following 6 weeks pharmacologic treatments was investigated using Yale Global Tic Severity Scale (YGTSS) during visits at weeks 0, 4, 8, and 12.

**Results:** Of 150 patients, 76% (114/150) were male (age range, 3.03–14.24 years; mean, 8.11 ± 2.48 years). Patients were divided into three groups: tiapride (*n* = 94), CAP (*n* = 14), and CAP + tiapride (*n* = 42). The mean YGTSS improved 11.02, 15.14, 11.13 points from baseline to posttreatment for tiapride, CAP, and CAP + tiapride, respectively, but variance analysis showed there was no significant difference in YGTSS related to different pharmacologic intervention during subsequent visits at weeks 4, 8, and 12. Repeated measure analysis showed there was no significant difference between different medication types for reducing the YGTSS score (*F* = 0.553, *P* = 0.576). No serious adverse events (AEs) occurred, and there was no significant difference in the prevalence of AEs between the three groups.

**Conclusion:** The CAP is effective and safe for TD management in a RWS, because of the limitation of sample size and the period of follow up, observational studies with longer-term outcomes, and larger sample size are needed.

## Introduction

Tic disorders (TDs) have been conceptualized as hyperkinetic-movement disorders and chronic neuropsychiatric disorder with childhood onset. TDs are characterized by multiple motor and one or more phonic tics; males are three-times more likely to suffer from TDs than females (1, 2). There are three types of TDs: transient tic disorder (TTD), chronic tic disorder (CTD), and Tourette syndrome (TS). One meta-analysis showed the worldwide prevalence of TTD to be 2.99%, followed by CTD (1.61%), and TS (0.77%) (3). In China, the combined prevalence of TDs has been reported to be 6.1%. One meta-analysis stated the prevalence of TTD, CTD, and TS in China to be 1.7, 1.2, and 0.3%, respectively (4). Despite the core symptom being tics, co-occurring psychiatric disorders are common in children suffering from TDs, such as attention deficit and hyperactivity disorder (ADHD), obsessive-compulsive disorder (OCD), and anxiety disorder (5–7).

Pharmacologic treatment is the main therapy for the management of tics and comorbidities (8, 9). Several randomized controlled trials (RCTs) have shown that clonidine can reduce some of the tics and other behavioral symptoms associated with TDs, and that clonidine is safe to use (10–12). In 2016, a systematic review by Wang and colleagues encompassing six RCTs (1,145 participants) evaluated the effectiveness of a clonidine adhesive patch (CAP) for TD treatment (13). They showed that the CAP may be as effective as haloperidol or tiapride for TDs, and that the prevalence of adverse events (AEs) of the CAP was low. Nevertheless, additional studies are needed urgently to reevaluate the effectiveness and safety of the CAP.

RCTs are undertaken according to regulatory and scientific standards, so extrapolation of research results is limited. Hence, RCTs may not necessarily reflect what happens in real-world settings (14). Real-world evidence helps to improve decision-making in healthcare settings. Hence, this study aimed to evaluate the effectiveness and safety of CAP for children with tic disorder (TD) in a real-world setting (RWS).

## Materials and Methods

### Study Design

This was an open-label, non-interventional, post-marketing, observational study to investigate the change in severity of tic symptoms in TD patients following different pharmacologic treatments in a real-world practice. The study was conducted from January to May 2019. The non-interventional, post-marketing nature of our study meant that registration in a clinical trial registry was not mandatory. Nevertheless, the study protocol was approved by the Office of Research Ethics Committees of West China Second Hospital (Chengdu, China).

### Inclusion Criteria

The inclusion criteria were: (i) a clinically confirmed diagnosis of TD according to the *Diagnostic and Statistical Manual of Mental Disorders-IV-Text Revision*; (ii) age <18 years; (iii) provision of written informed consent.

### Exclusion Criteria

The exclusion criteria were: (i) delays or problems with mental development (Wechsler intelligence-quotient score <70 points); (ii) cerebral palsy, neurodevelopmental delay, history of inherited metabolism, or poor development of motor language; (iii) non-provision of written informed consent. Voluntary written informed consent was provided by all caregivers or children aged >8 years.

### Treatment

Assignment of patients to pharmacologic therapy was decided according to standard practice and medical indications assessed by the treating physician. Eligible patients were prescribed a drug dose by their treating physician depending on its effectiveness and toxicity and patient weight. This dosing regimen conformed to standard medical practices and the terms of local marketing authorization and reimbursement guidelines.

For CAPs, patients of weight 20–40 kg were given 1.0 mg/film; 40–60 kg were given 1.5 mg/film; >60 kg were given 2.0 mg/film. For tiapride, each child was started on 50 mg/day and the dose increased gradually to a maximum of 400 mg/day. The stable dose of medication was maintained for 6 weeks.

### Assessments

**Figure 1:**
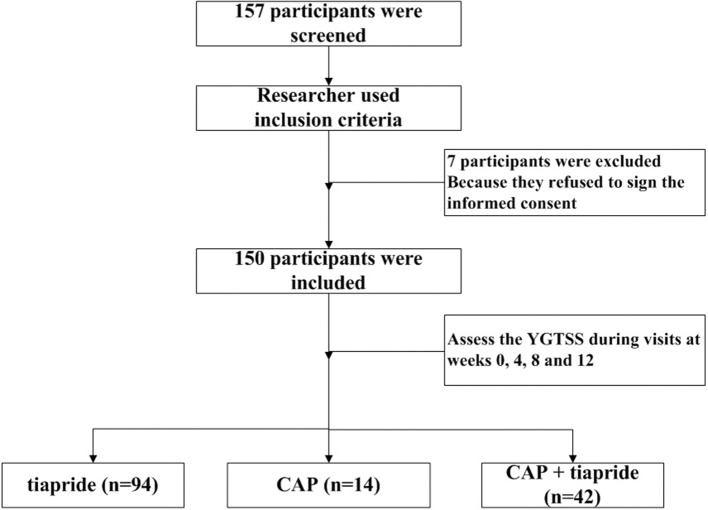
The study flow of screening the participants.

Baseline data (age, sex, disorder duration, type of TD) were obtained by the treating physician, followed by assessments during subsequent visits at weeks 0, 4, 8, and 12. The primary outcome was measured by the Yale Global Tic Severity Scale (YGTSS). This consists of a separate rating of severity for motor and vocal tics along five discriminant dimensions (number, frequency, intensity, complexity, and interference) on a scale of 0–5 for each. Summation of these scores (i.e., 0–50) results in a total tic score (TTS). The tic impairment score (TIS), from zero to a maximum of 50 points, is based on the impact of the tic disorder on self-esteem, family life, social acceptance, and school performance. The TIS is added to the TTS to obtain the total YGTSS (YGTSS-T) score. The secondary outcome was AEs, which were assessed via an interview of self-report by participants or their caregivers during the follow-up. The study flow of screening the participants was shown in Figure 1.

### Statistical Analyses

The intent-to-treat population (which comprised all patients who received the study drug and who had a baseline visit and at least one on-treatment post-baseline visit) was used to assess all effectiveness and safety variables. For the comparability of baseline variables with continuous variables, they are shown as the mean ± standard deviation. If data followed a normal distribution, one-way ANOVA was used. If data did not follow a normal distribution, they were analyzed by the Wilcoxon rank sum test. Categorical variables are shown as frequencies and percentages, and the chi-square test or Fisher's exact test were used as appropriate.

Repeated measure analysis of variance was used to test the difference of treatment effects for different drugs. First, we used the Mauchly sphericity test to ascertain if the measured data satisfied the test hypothesis. If not, the Greenhouse–Geisser correction was carried out. *P* ≤ 0.05 was considered significant. We used SPSS v22 (IBM, Armonk, NY, USA) for statistical analyses.

## Results

### Characteristics of Patients (Table 1)

Children diagnosed with TDs were enrolled from a pediatric neurology clinic at West China Second Hospital. Initially, 157 patients were recruited. Seven patients discontinued treatment because they refused to sign the informed consent, so 150 patients completed the study. Among these 150 children, 76% (114/150) were male, and ranged in age from 3.03 years to 14.24 (mean, 8.11 ± 2.48) years. These patients were divided into three intervention groups: tiapride (*n* = 94), CAP (*n* = 14), and CAP + tiapride (*n* = 42).

**Table 1:** Demographics and baseline patient characteristic.

**Group**		**Tiapride (*n* = 94)**	**Clonidine (*n* = 14)**	**Clonidine + tiapride (*n* = 42)**	**χ^2^/F**	***P***
Age		7.958 ± 2.499	6.965 ± 2.341	8.822 ± 2.338	3.507	0.033
Gender	Male	67	13	34	4.483	0.106
	Female	27	1	8		
Time of disorder (year)		1.555 ± 1.451	1.001 ± 1.138	1.750 ± 1.613	1.358	0.260
Type of TDs	TTD	36	9	15	3.969	0.410
	CTD	35	3	15		
	TS	23	2	12		
YGTSS score	Total scores	24.800 ± 7.003	28.00 ± 13.801	27.64 ± 10.727	1.898	0.154
	Motor tic	10.320 ± 3.303	10.640 ± 3.875	10.500 ± 3.624	0.078	0.925
	Vocal tic	3.970 ± 4.723	4.500 ± 5.095	5.480 ± 5.260	1.369	0.258
	Tic impairment score	10.530 ± 4.242	12.860 ± 8.254	11.670 ± 5.372	1.706	0.185

There was no significant difference between the three groups in terms of sex, disorder duration, type of TD, or baseline YGTSS score, but there was a significant difference in age, so we included age as a covariate in the statistical model for analyses.

### Effectiveness of Different Pharmacologic Interventions

The YGTSS score was reduced at different times after pharmacologic intervention. For tiapride, the mean YGTSS improved 11.02 points from baseline to posttreatment (24.8 vs. 13.78). For CAP, the mean YGTSS improved 15.14 points from baseline to posttreatment (28.0 vs. 12.86). For CAP + tiapride, the mean YGTSS improved 11.13 points from baseline to posttreatment (27.64 vs. 16.31), but variance analysis showed there was no significant difference in YGTSS related to different pharmacologic intervention during subsequent visits at weeks 4, 8, and 12 (Table 2).

**Table 2:** YGTSS scores for different treatment times in three groups.

	**Baseline**	**4 weeks**	**8 weeks**	**12 weeks**
Tiapride	24.800 ± 7.003	21.050 ± 9.141	18.300 ± 9.562	13.780 ± 11.577
Clonidine	28.00 ± 13.801	17.860 ± 10.037	16.360 ± 12.768	12.860 ± 12.805
Clonidine + tiapride	27.64 ± 10.727	23.140 ± 12.233	20.710 ± 13.349	16.310 ± 14.338
*F*	1.89	1.517	1.071	0.712
*P*	0.154	0.223	0.345	0.492

Repeated measure analysis also showed a significant difference for the YGTSS score for different medications at different follow-up times (*F* = 18.949, *P* = 0.000), but we found no interaction between the medication time and medication type (*F* = 1.043, *P* = 0.389) or between the medication time and age (*F* = 2.384, *P* = 0.069). A test of within-participant effects showed no significant difference between different medication types for reducing the YGTSS score (*F* = 0.553, *P* = 0.576).

### Safety

We found that 10.6% (10/94) of patients reported AEs in the tiapride group (Table 3); the most common AEs were dizziness and abdominal pain. Also, 7.1% (1/14) of patients reported AEs in the CAP group; the most common AEs were rash. In addition, 23.1% (10/42) of patients reported AEs in the CAP + tiapride group; the most common AEs were dizziness, abdominal pain, and drowsiness. The chi-square test showed no significant difference in the prevalence of AEs between the three groups.

**Table 3:** Reported side effects during follow up period.

	**Tiapride**	**Clonidine**	**Clonidine + tiapride**
Dizzy	3	0	4
Abdominal pain	2	0	2
Drowsiness	1	0	2
Insomnia	1	0	0
Nausea/vomit	1	0	0
Loss of appetite	1	0	0
Rash	1	1	0
Mental distress	0	0	2
Total	10	1	10

## Discussion

We used an observational study design to investigate the effectiveness and safety of different pharmacologic treatments in a real-world practice. We compared three pharmacologic interventions (tiapride, CAP, tiapride+ CAP) in 150 patients with TDs. We observed no significant difference between the medication groups in terms of reducing the YGTSS score after 8 weeks of treatment. In general, tiapride and the CAP were well-tolerated, and elicited few AEs.

Clonidine use is strongly recommended in Canadian guidelines, and it is regarded as first-line treatment in Chinese clinical guidelines, for TDs (15, 16). However, European guidelines recommend clonidine as second-line treatment for TDs (antipsychotic agents are first-line treatment) (17). Clonidine is seldom chosen to treat TDs in Japan (but clonidine is the only α2 adrenergic receptor agonist used to treat hypertension in Japan) (18). North American guidelines recommend α2 adrenergic agonists for the treatment of tics if the benefits of treatment outweigh the risks (with evidence level B), but physicians should monitor common side effects, such as sedation, heart rate, and blood pressure (level-A evidence) (19). Hence, the safety of the medication is an important concern for long-term treatment of TDs. We did not find serious AEs in TD treatment in a real-world practice during a short follow-up period, but common side effects must be monitored.

No clinical guidelines have mentioned CAP use, and there may be two reasons for this. First, formulations applied as an adhesive patch are not used widely. Nevertheless, a CAP releases clonidine at a relatively invariable rate for 1 week without trough or peak changes in plasma concentrations, so it is a very convenient treatment. Second, adhesive patches are relatively expensive, but the convenience of treatment may improve the quality of life of patients with TDs. Hence, cost–benefit analyses should be carried out. Therefore, future research should be considered from these two perspectives.

Our findings are similar to the study conducted by Joo and Kim (20), they assessed the effectiveness and safety of clonidine extended release (ER) treatment in Korean youth with ADHD and/or TS and included 29 children treated with clonidine ER, the result showed significant decreases in the CGI-S scores for both ADHD and tic symptoms were noted over 12 weeks, and life-threatening adverse effects were not observed.

Our study had three main limitations. First, included patients were from a single center, so a selection bias was evident. Nevertheless, the West China Second Hospital is the largest hospital in western China, so the research results carry a certain representativeness. Second, the observational, non-interventional design of our study may have introduced some bias toward overestimation of the treatment effect. Nevertheless, our study provides important insights into the real-world management of TDs. Finally, an observational study may underestimate AEs because adverse events were only assessed via an interview of self-report by participants or their caregivers, sometimes they may ignore reporting adverse reactions. Further studies are needed to overcome the shortcomings mentioned above.

## Conclusions

The CAP is effective and safe for TD management in a RWS, because of the limitation of sample size and the period of follow up, observational studies with longer-term outcomes and larger sample size are needed.

## Data Availability Statement

The raw data supporting the conclusions of this article will be made available by the authors, without undue reservation, to any qualified researcher.

## Ethics Statement

The studies involving human participants were reviewed and approved by the Office of Research Ethics Committees of West China Second Hospital. Written informed consent to participate in this study was provided by the participants' legal guardian/next of kin. Written informed consent was obtained from the individual(s), and minor(s)' legal guardian/next of kin, for the publication of any potentially identifiable images or data included in this article.

## Author Contributions

CY designed the review, collected data, carried out analysis and interpretation of the data, and wrote the review. BK and DY designed the review, collected data, checked the data, and wrote the review. LZhao and LZhan designed the review, commented on drafts for previous version.

## Conflict of Interest

The authors declare that the research was conducted in the absence of any commercial or financial relationships that could be construed as a potential conflict of interest.
